# Verbesserte radiologische Darstellung kongenitaler Auralatresien mittels „flat-panel volume CT“

**DOI:** 10.1007/s00106-024-01511-1

**Published:** 2024-09-16

**Authors:** Franz-Tassilo Müller-Graff, Jan von Düring, Johannes Voelker, Fadi Al-Tinawi, Rudolf Hagen, Tilmann Neun, Stephan Hackenberg, Kristen Rak

**Affiliations:** 1 Klinik und Poliklinik für Hals‑, Nasen- und Ohrenkrankheiten, plastische und ästhetische Operationen und das Comprehensive Hearing Center, Universitätsklinikum Würzburg, Josef-Schneider-Straße 11, 97080 Würzburg, Deutschland; 2 Institut für Diagnostische und Interventionelle Neuroradiologie, Universitätsklinikum Würzburg, Würzburg, Deutschland

**Keywords:** OTOPLAN, Computertomographie basierte Software, Atresieplatte und Nervus facialis, Ohrmissbildung, Mittelohrfehlbildung, OTOPLAN, Computed-tomography-based software, Atresia plate and facial nerve, Ear deformity, Middle ear malformation

## Abstract

**Hintergrund:**

Eine präzise präoperative radiologische Evaluation von Auralatresien ist für die Operationsplanung von größter Bedeutung. Bisher wurde hierfür die Multislice-Computertomographie (MSCT) verwendet, die jedoch kleine Strukturen wie den Stapes nur unzureichend dargestellen kann. Die „flat-panel volume CT“ (fpVCT) mit ihren sekundären Rekonstruktionen (fpVCT_SECO_) bietet eine hochauflösende Darstellung des Mittelohrs. Eine neue otochirurgische Planungssoftware ermöglicht überdies die detaillierte 3‑D-Rekonstruktion der Mittelohranatomie.

**Ziel der Arbeit:**

Evaluierung des Einsatzes von fpVCT_SECO_ in Kombination mit einer otochirurgischen Planungssoftware zur genaueren Diagnose und Behandlung kongenitaler Auralatresien.

**Material und Methoden:**

Sieben Patienten mit kongenitaler Auralatresie erhielten präoperativ ein MSCT (600 µm Schichtdicke) und ein korrespondierendes fpVCT (466 µm Schichtdicke). Zusätzlich wurden fpVCT_*SECO*_ (99 µm Schichtdicke) rekonstruiert. Die Jahrsdoerfer- und Siegert-Grading-Scores wurden bestimmt und ihre Anwendbarkeit in den oben genannten bildgebenden Modalitäten bewertet. Zusätzlich wurde der Malleus-incus-Komplex im 3‑D-Rendering analysiert.

**Ergebnisse:**

Die Bildgebung mit fpVCT_*SECO*_ ermöglichte eine zuverlässige Darstellung der Anomalien, insbesondere der Gehörknöchelchenkette. Es wurde ein signifikanter Unterschied im Siegert-Grading-Score festgestellt. Zusätzlich konnte der Malleus-Incus-Komplex in 3‑D besser dargestellt werden.

**Diskussion:**

Die Einführung neuer bildgebender Verfahren und chirurgischer Planungstechniken in das diagnostische Konzept der Auralatresie erleichtert die Identifizierung der fehlgebildeten Anatomie und ermöglicht eine systematische Analyse. Diese Kombination kann auch dazu beitragen, die Pathologie genauer zu klassifizieren und damit die Sicherheit und den Erfolg des chirurgischen Eingriffs zu erhöhen.

## Abstract (en)

**Background:**

Precise preoperative radiological evaluation of aural atresia is of utmost importance for surgical planning. Until now, multislice computed tomography (MSCT) has been used but it cannot adequately visualize small structures such as the stapes. Flat-panel volume CT (fpVCT) with its secondary reconstructions (fpVCT_SECO_) offers a high-resolution visualization of the middle ear. New otosurgical planning software also enables detailed 3D reconstruction of the middle ear anatomy.

**Aim of the work:**

Evaluation of the use of fpVCT_SECO_ in combination with an otosurgical planning software for a more accurate diagnosis and treatment of congenital aural atresia.

**Material and methods:**

Seven patients with congenital aural atresia underwent preoperative MSCT (600 µm slice thickness) and corresponding fpVCT (466 µm slice thickness). In addition, fpVCT_*SECO*_ (99 µm slice thickness) were reconstructed. The Jahrsdoerfer and Siegert grading scores were determined and their applicability in the abovementioned imaging modalities was evaluated. In addition, the malleus incus complex was analyzed in 3D rendering.

**Results:**

Imaging with fpVCT_*SECO*_ enabled reliable visualization of the abnormalities, in particular the ossicular chain. A significant difference in the Siegert grading score was found. In addition, the malleus-incus complex could be visualized better in 3D.

**Discussion:**

The introduction of new imaging techniques and surgical planning techniques into the diagnostic concept of aural atresia facilitates the identification of malformed anatomy and enables systematic analysis. This combination can also help to more accurately classify the pathology and thus increase the safety and success of the surgical procedure.

Kongenitale Auralatresien sind seltene angeborene Fehlbildungen, die für die Betroffenen mit funktionellen und kosmetischen Einschränkungen verbunden sind. Eine exakte radiologische Darstellung dieser Pathologien ist mit herkömmlichen bildgebenden Verfahren aus technischen Gründen oft nur unzureichend möglich. Neue radiologische Techniken und computergestützte Software sind vielversprechende Entwicklungen, insbesondere für Kinder, bei denen eine optimale Versorgung im Hinblick auf eine mögliche chirurgische Hörrekonstruktion gewährleistet sein sollte.

Die kongenitale Auralatresie ist durch eine Hypo- oder Aplasie des äußeren Gehörgangs in Verbindung mit einer dysmorphen Ohrmuschel gekennzeichnet. Außerdem sind das Mittelohr und insbesondere die Gehörknöchelchen sowie gelegentlich auch das Innenohr fehlgebildet [19]. Die geschätzte Inzidenz der Auralatresie wird auf etwa 1 von 11.000 bis 15.000 Neugeborenen angegeben [17]. Die Auralatresie kann isoliert oder im Rahmen eines Syndroms auftreten [4, 23].

Bildgebende Verfahren sind für die Beurteilung des Schweregrades der klinischen Diagnose einer Auralatresie und für die präoperative Beurteilung eines möglichen chirurgischen Eingriffs unerlässlich. Derzeit wird die CT(Computertomographie)-Bildgebung als diagnostische Methode der Wahl empfohlen, da sie dem Chirurgen hilft, die Anatomie des Mittelohrs vorherzusagen und eine prognostische Grundlage für den chirurgischen Eingriff bietet [23].

Zur genaueren Einteilung der Auralatresie aus chirurgischer Sicht haben Jahrsdoerfer et al. einen radiologischen Grading-Score beschrieben, der in der klinischen Praxis häufig verwendet wird [8]. Dieser Score kann bei der Auswahl der Patienten helfen, die eine realistische Chance auf eine klassische chirurgische Wiederherstellung des Hörens haben. Siegert et al. erweiterten und ergänzten diese Klassifikation durch ein semiquantitatives System zur Einschätzung der Operationsindikationen [22]. Mit der konventionellen Multislice-CT (MSCT) können jedoch kleine Strukturen, wie der Steigbügel, die für die Klassifizierung und auch für den Operationserfolg sehr wichtig sind, nicht ausreichend dargestellt werden. Die „flat-panel volume computed tomography“ (fpVCT) ist eine neue CT-Technik, die eine Darstellung mit sehr hoher räumlicher Auflösung ermöglicht. In mehreren Studien konnte eine signifikant bessere Darstellung der feinen knöchernen Strukturen des Mittelohres und des Felsenbeins [9] im Vergleich zur MSCT sowie eine Reduktion der effektiven Dosis um ca. 40 % im Vergleich zur 64-Schichten-MSCT [6]- und 128-Schichten-MSCT nachgewiesen werden [16].

Darüber hinaus bietet die fpVCT durch ihre sekundären Rekonstruktionen (fpVCT_SECO_) die Möglichkeit, die Bildqualität und räumliche Auflösung ohne zusätzliche Strahlenbelastung weiter zu verbessern [15]. Soweit uns bekannt ist, war diese Technik noch nie Gegenstand von Untersuchungen von Auralatresien. Hierbei handelt es sich um eine Verkleinerung des „volume of interest“ (VOI) bei gleichbleibender Matrix, wodurch die Schichtdicke verkleinert und die Auflösung innerhalb des VOI erhöht wird. Hilfreich für die weiterführende Bildverarbeitung ist, dass die fpVCT-Technik isotrope Voxel mit gleicher Kantenlänge erzeugt. Im Vergleich dazu erzeugt die MSCT anisotrope Voxel mit ungleicher Kantenlänge. Die Begrifflichkeit „sekundäre Rekonstruktion“ geht auf den Erstbeschreiber dieses Verfahrens zurück und betont damit, dass die Veränderungen des VOI und damit der Schichtdicke nach der Rekonstruktion der Schichtbilder aus dem primären Datensatz erfolgt [15].

Die neu entwickelte otologische Planungssoftware OTOPLAN® (CAScination, Bern, Schweiz, in Zusammenarbeit mit MED-EL, Innsbruck, Österreich) ist eine Tablet-basierte Software, die für die präoperative Planung von otologischen Eingriffen konzipiert wurde [7, 12, 13]. Eine der Hauptfunktionen ist die Visualisierung der einzelnen Mittelohrstrukturen, die eine Darstellung der Bilddaten in den 3 Körperachsen und eine 3‑D-Visualisierung durch automatisierte Segmentierung ermöglicht, die einen Vergleich der verschiedenen Bildgebungsmodalitäten erlaubt [3].

Diese digitalen Werkzeuge bieten dem Chirurgen eine große Hilfe bei der präoperativen Planung, insbesondere bei jungen Patienten, die aufgrund ihrer Entwicklung von einem maximalen Hörgewinn profitieren. Bisher wurden mit dieser Software nur Fehlbildungen des Innenohrs untersucht [2], jedoch keine Fälle von Auralatresien.

Ziel dieser Studie war es zu untersuchen, ob die fpVCT (466 µm Schichtdicke) und insbesondere deren sekundäre Rekonstruktionen (fpVCT_SECO_ – 99 µm Schichtdicke) die radiologische Diagnostik der kongenitalen Auralatresie im Vergleich zur konventionellen MSCT (600 µm Schichtdicke) erleichtern. Um diese Frage zu beantworten, wurde 1. untersucht, ob sich die radiologischen Grading-Scores nach Jahrsdoerfer und Siegert bei Verwendung der unterschiedlichen Bildgebungsmodalitäten unterscheiden. Weiterhin wurde 2. die Eignung der fpVCT_SECO_ in Kombination mit der neuen otologische Planungssoftware für die Diagnose der kongenitalen Atresie evaluiert. Schließlich 3. wurde die 3D-Darstellung des Malleus-Incus-Komplexes in der otologische Planungssoftware untersucht.

## Methoden

### Probanden

In dieser retrospektiven Studie wurden 7 Felsenbein-Scans von Patienten mit kongenitaler Auralatresie (6 unilateral, 1 bilateral) untersucht, die im Rahmen der präoperativen Diagnostik durchgeführt wurden. In die Studie wurden ausschließlich Patienten aufgenommen, die sowohl eine MSCT- als auch eine fpVCT-Untersuchung durchlaufen hatten. In unserem Haus verwenden wir bei allen perioperativen Planungen von Hörimplantaten standardmäßig ein fpVCT mit sekundären Rekonstruktionen aufgrund der hohen Auflösung. Wurde zuvor bereits ein Felsenbein-CT, meist in einer externen Einrichtung, durchgeführt, so wurde dieses für die retrospektive Datenanalyse der Studie herangezogen. Aus den Daten der fpVCT-Scans wurden sekundäre Rekonstruktionen (fpVCT_SECO_) erstellt. Das Alter der Patienten zum Zeitpunkt der Bildgebung lag zwischen 15 und 59 Jahren (Mittelwert 35, Median 31 Jahre). 4 Ohren mit Auralatresie waren linksseitig, 3 rechtsseitig. Die Mittelohrstrukturen wurden mit der otologischen Planungssoftware OTOPLAN® (CAScination, Bern, Schweiz, in Zusammenarbeit mit MED-EL, Innsbruck, Österreich) dargestellt. Als Kontrolle dienten Scans der nicht betroffenen kontralateralen Seite der 6 einseitig auralatretischen Patienten verwendet.

Diese retrospektive, anonymisierte Studie wurde in Übereinstimmung mit den lokalen Richtlinien und den Prinzipien der Deklaration von Helsinki und der Guten Klinischen Praxis (Good Clinical Practice) durchgeführt und von der lokalen Ethikkommission genehmigt (2021050601).

### Bildgebung

Die MSCT-Datensätze wurden an den MSCT-Scannern „SOMATOM Definition AS+“ (Siemens), „Brilliance 40“ (Philips), „Alexion“ (Toshiba) und „Aquilion Prime“ (Toshiba) mit einer medianen Schichtdicke von 600 µm aufgenommen. Die Werte der Röhrenparameter betrugen im Median: Röhrenstrom („tube current“): 75 mA; Röhrenspannung („tube voltage“): 114 kV. Die Untersuchungszeit war, sofern in den externen Bildgebungen angegeben, im Median 3,7 s.

FpVCT-Scans wurden mit einem Angiographiegerät (Axiom Artis; Siemens Healthcare AG, Erlangen, Deutschland) mit kommerziell erhältlicher Software (Syngo DynaCT, Siemens) durchgeführt. Die Datensätze wurden mit den folgenden medianen Parametern akquiriert: 20s DCT Head protocol; Röhrenstrom = 21 mA; Röhrenspannung = 109 kV; Rotationswinkel („rotation angle“) = 200°; Pulslänge („pulse length“) = 3,5 ms; Bildwinkelschritt („frame angulation step“) = 0,5°/Bild; mittlere Schichtdicke = 466 µm. Aus diesen Datensätzen wurde die fpVCT_SECO_ nach den Erkenntnissen von Pearl et al. mit den folgenden Einstellungen generiert: 512 × 512 Schnittmatrix; HU-Kernel-Typen („Hounsfield unit kernel types“); scharfe Bildmerkmale („sharp“); mediane Schichtdicke = 99 µm [15].

### Bewertung verschiedener Bildgebungsmodalitäten zur Untersuchung von Auralatresien

Zur Beurteilung der kongenitalen Auralatresien wurden die Datensätze über das PACS(Picture Archiving and Communication System)-Netzwerk des Krankenhauses in den DICOM(Digital Imaging and Communications in Medicine)-Standard konvertiert. Anschließend wurden sie anonymisiert in die otologische Planungssoftware übertragen und die Jahrsdoerfer- und Siegert-Grading-Scores ermittelt. Alle Strukturen, die für die Bestimmung dieser Scores identifiziert wurden, sind in Tab. 1 aufgeführt.

**Tab. 1:** Gliederung der Grading-Scores der Auralatresie

Struktur	Jahrsdoerfer-Score	Siegert-Score
*Konfiguration*	*Vorhanden/nicht vorhanden [Punkte]*	*Normal/leicht dysplastisch/schwer dysplastisch [Punkte]*
Stapes	2/0	4/2/0
Malleus-Incus-Komplex	1/0	2/1/0
Art. incudostapedialis	1/0	–
N. facialis	1/0	4/2/0
Rundes Fenster	1/0	4/0
Ovales Fenster	1/0	40
Mastoid-Pneumatisation	1/0	2/1/0
Meatus acusticus externus	1/0	2/1/0
Größe und Belüftung des Mittelohrs	1/0	4/2/0
Verlauf der Arterien und Venen	–	2/1/0
Maximale Punktzahl	*10*	*28*

Die verschiedenen anatomischen Teilstrukturen der Gehörknöchelchen, ob normal oder fehlgebildet, wurden anschließend mit einer modifizierten Skala und Bewertungsliste, wie von Majdani et al. beschrieben, anatomisch genauer untersucht [9], was zu einem erweiterten Score, dem „ossicle score“, führte. Dabei wurde die Abgrenzung zum umgebenden Gewebe anhand einer numerischen Skala von 0–3 bewertet. 0: die Struktur wurde nicht erkannt, 1: die Struktur konnte nicht leicht von den umgebenden Strukturen abgegrenzt werden, 2: die Struktur war mäßig abgegrenzt und 3: die Struktur war gut vom umgebenden Gewebe abgegrenzt. Auf der Grundlage dieser Bewertungsskala wurde die Identifizierung der verschiedenen Strukturen in Prozentwerten berechnet.

Die Visualisierung des Malleus-Incus-Komplexes mit den verschiedenen Bildgebungsmodalitäten erfolgte mit dem automatischen Segmentierungswerkzeug der Planungssoftware. Basierend auf der individuellen Auswahl eines einzelnen Punktes um das Incudomalleolar-Gelenk durch den Benutzer wurde ein kombiniertes Segment für den Komplex erstellt.

Der Vergleich des Jahrsdoerfer- und des Siegert-Scores wurde für alle Probanden zwischen den 3 Bildgebungsmodalitäten durchgeführt. Die Analyse wurde in einer Testreihe von 2 verschiedenen Untersuchern mit großer Erfahrung in der Beurteilung von Felsenbeinbildgebungen durchgeführt: leitender HNO-Oberarzt (KR) und HNO-Facharzt (FTMG) mit Schwerpunkt Otologie, Supervision durch neuroradiologischen Facharzt (TN). Die Patientendaten wurden anonymisiert. Die Reihenfolge der Auswertung wurde auf 3 Arten randomisiert: Randomisierung der Bildgebungsmodalität, Randomisierung der Felsenbeine und Randomisierung der Score-Strukturen.

### Statistik

Vor den parametrischen Analysen wurde die Normalverteilung aller Datenreihen durch Kolmogorov-Smirnov bestätigt. Die einfaktorielle Varianzanalyse mit wiederholten Messungen (ANOVA) wurde angewandt, um die Unterschiede in den absoluten Mittelwerten der Grading-Scores in den 3 Modalitäten und Einstellungen zu bewerten. Für Mehrfachvergleiche wurde der Tukey-Test für Mehrfachvergleiche verwendet. Unterschiede mit einem *p*-Wert von weniger als 0,05 wurden als statistisch signifikant angesehen.

Die statistische Analyse und die Erstellung von Diagrammen erfolgte mit GraphPad Prism (Version 8.4.0, San Diego, CA, USA) und IBM SPSS Statistics (Version 25.0.0.0; IBM Corporation, Armonk, NY, USA). Die Daten werden in Balkendiagrammen dargestellt.

## Ergebnisse

### Mittelohrstrukturen

Um den Grad der Mittelohrpathologie bei Auralatresie zu beurteilen, wurde bei allen Patienten eine radiologische Diagnostik des Felsenbeins durchgeführt. Aus den Bilddaten wurden Strukturen visualisiert, die sowohl zum Jahrsdoerfer- als auch zum Siegert-Score beitragen. Dazu gehören die fehlgebildeten Gehörknöchelchen, Abweichungen im Verlauf des N. facialis, Konfigurationen des ovalen und runden Fensters sowie die Pneumatisierung der Mastoidzellen und der Gehörgänge. Alle Strukturen konnten mit der fpVCT_SECO_ im Vergleich zur MSCT hinsichtlich ihrer Anatomie und Integrität genauer dargestellt werden. Im Folgenden werden ausgewählte Strukturen näher beschrieben.

#### Stapes.

**Abb. 1:**
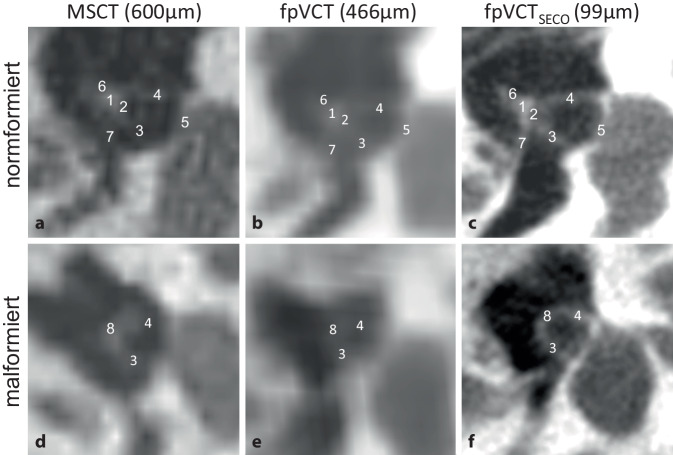
Darstellung des Stapes mit verschiedenen Bildgebungsmodalitäten und -einstellungen. Repräsentative Bilder eines normformierten Stapes mit MSCT (600 µm Schichtdicke) sind in **a**, fpVCT (466 µm Schichtdicke) in **b** und fpVCT_SECO_ (99 µm Schichtdicke) in **c** dargestellt. **d**–**f** zeigen die Ansicht des Stapes bei Fehlbildungen. Folgende Substrukturen des Stapes wurden identifiziert: *1* Caput stapedis, *2* Collum stapedis, *3* Crus anterius stapedis, *4* Crus posterius stapedis, *5* Basis stapedis, *6* Art. incudostapedialis, *7* M. stapedius, *8* fehlgebildeter Stapes. *MSCT* Multislice-Computertomographie, *fpVCT* „flat-panel volume computed tomography“, *fpVCT*_*SECO*_ sekundäre Rekonstruktionen der fpVCT

Der Stapes konnte mit fpVCT_SECO_ sehr präzise dargestellt werden. Statt der nur schemenhaften Darstellung mit MSCT zeigte die fpVCT_SECO_ Details verschiedener Substrukturen wie Caput und Collum. Außerdem wurden beide Crura, sofern vorhanden, vollständig dargestellt und in vielen Fällen auch die Fußplatte (Basis stapedis) erkannt. In exzellenten Bildbeispielen konnte auch der M. stapedius identifiziert werden. Als wichtige Verbindung zum Malleus-Incus-Komplex konnte auch das Incudostapedialgelenk mit der fpVCT_SECO_ deutlicher dargestellt werden. Ein Beispiel für einen normalen und einen fehlgebildeten Stapes in den verschiedenen Bildgebungsmodalitäten ist in Abb. 1 zu sehen.

#### Malleus-Incus-Komplex.

**Abb. 2:**
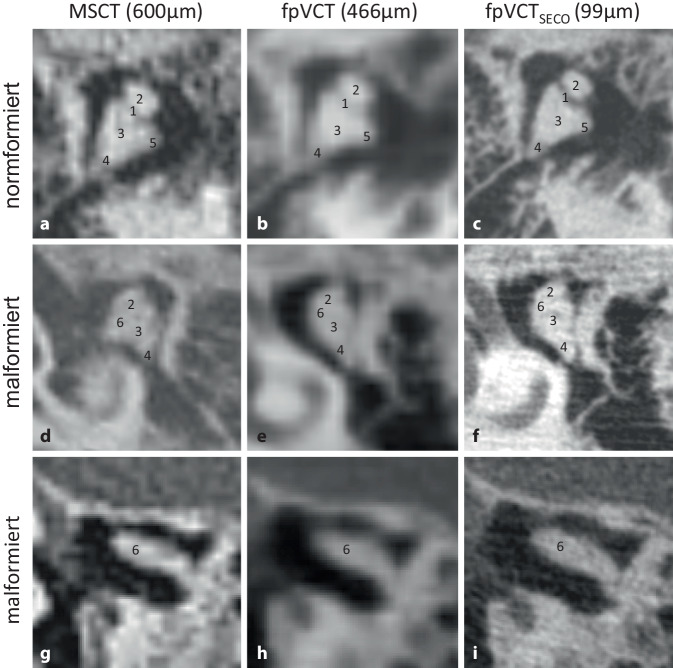
Darstellung des Malleus-Incus-Komplexes mit verschiedenen Bildgebungsmodalitäten und -einstellungen. Ein repräsentatives Bild von MSCT (600 µm Schichtdicke) ist in **a**, von fpVCT (466 µm Schichtdicke) in **b** und von fpVCT_SECO_ (99 µm Schichtdicke) in **c** gezeigt. **d**–**f** zeigen den Malleus-Incus-Komplex mit leichten Fehlbildungen, **g**–**i** mit schweren Fehlbildungen. Die identifizierten Substrukturen des Malleus-Incus-Komplexes waren *1* Art. incudomalleolaris, *2* Caput mallei, *3* Corpus incudis, *4* Crus breve incudis, *5* Crus longum incudis, *6* verschmolzenes Art. incudomalleolaris. *MSCT* Multislice-Computertomographie, *fpVCT „*flat-panel-volume computed tomography“, *fpVCT*_*SECO*_ sekundäre Rekonstruktionen der fpVCT

Ein Beispiel für einen regulären, leicht fehlgebildeten und stark fehlgebildeten Komplex in den verschiedenen Bildgebungsmodalitäten ist in Abb. 2 dargestellt. Obwohl die Articulatio incudomalleolaris in einigen MSCT-Datensätzen abgegrenzt wurde, war eine zuverlässige Bestimmung nur mit der fpVCT_SECO_ möglich. In der niedrig auflösenden Bildgebung war es daher oft nicht möglich, eindeutig zu unterscheiden, ob das Gelenk fusioniert oder nur radiologisch schlecht dargestellt war.

Die Ergebnisse des „ossicle score“ sind in Tab. 2 dargestellt. Sowohl die Stapes-Strukturen als auch die Strukturen des Art. incudostapedialis und des Malleus-Incus-Komplexes wurden mit der fpVCT_SECO_ mit höchsten Prozentsätzen identifiziert.

**Tab. 2:** „Ossiclescore“. Ergebnisse (%) für die Identifizierung der anatomischen Strukturen der Gehörknöchelchen, sofern vorhanden, basierend auf dem „ossicle score“ bei Patienten mit Auralatresie

	MSCT	fpVCT	fpVCT_SECO_
*Patienten*	7	7	7
*Nummer des Bewerters*	2	2	2
–	*Identifizierung (%)*	*Identifizierung (%)*	*Identifizierung (%)*
*Stapes*			
Caput	45,83	54,17	83,33
Collum	20,83	37,50	75,00
Crus anterior	30,00	36,67	70,00
Crus posterior	13,33	40,00	70,00
Basis stapedis	26,67	60,00	76,67
*Mittelwert*	*27,33*	*45,67*	*75,00*
*Incus/Stapes*			
Art. incudostapedialis	*25,00*	*33,33*	*83,33*
*Malleus/Incus*			
Malleus-Incus-Komplex	86,67	90,00	100,00
Corpus incudes	86,67	80,00	100,00
Caput mallei	80,00	80,00	100,00
Art. incudomalleolares	37,50	44,44	87,50
Crus breve incudis	37,33	73,33	100,00
Crus longum incudis	58,33	70,37	100,00
*Mittelwert*	*70,42*	*73,03*	*97,92*

*MSCT* Multislice-Computertomographie, *fpVCT „*flat-panel-volume computed tomography“, *fpVCT*_*SECO*_ sekundäre Rekonstruktionen der fpVCT

#### N. facialis.

**Abb. 3:**
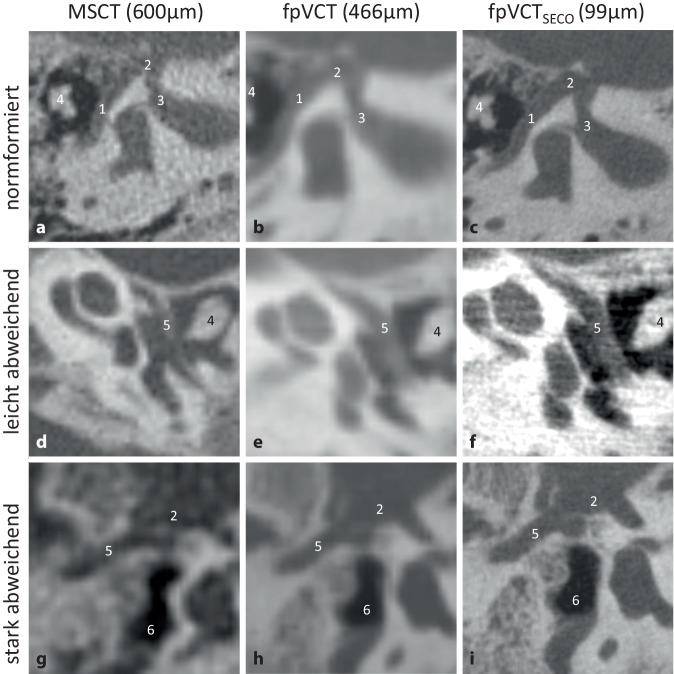
Visualisierung des Verlaufs des Nervus facialis. Ein repräsentatives Bild mit MSCT (600 µm Schichtdicke) ist in **a**, eine Darstellung von fpVCT (466 µm Schichtdicke) in **b** und fpVCT_SECO_ (99 µm Schichtdicke) in **c** gezeigt. **d**–**f** zeigen einen leicht abweichenden Verlauf des N. facialis durch die Paukenhöhle. **g**–**i** zeigen einen stark abweichenden Verlauf des N. facialis. *1* Canalis nervi facialis, *2* Ganglion geniculatum, *3* Meatus acusticus internus, *4* Malleus-Incus-Komplex, *5* dysmorpher Verlauf des N. facialis, *6* dysmorphe verkleinerte Paukenhöhle. *MSCT* Multislice-Computertomographie, *fpVCT* „flat-panel-volume computed tomography“, *fpVCT*_*SECO*_ sekundäre Rekonstruktionen der fpVCT

Von den drei untersuchten Bildgebungsmodalitäten lieferte das fpVCT_SECO_ die genaueste Darstellung des N. facialis, was für das chirurgische Vorgehen entscheidend ist. Insbesondere war eine bessere Abgrenzung bei abweichendem Verlauf des Nervs durch die Paukenhöhle erkennbar. Repräsentative Bilder eines normalen sowie eines leicht und stark abweichenden Verlaufs des N. facialis sind in Abb. 3 dargestellt.

#### Ovales und rundes Fenster.

Der Ausschluss einer Ossifikation der cochleären Fenster war im Allgemeinen mit allen 3 Bildgebungsmodalitäten möglich. Wenn jedoch die Bildgebung mit niedriger Auflösung eine ungünstige Schnittebene zeigte, konnte der Eindruck eines verknöcherten Fensters entstehen, das sich in der höheren Auflösung als offen erwies.

#### Mastoidpneumatisierung und äußerer Gehörgang.

**Abb. 4:**
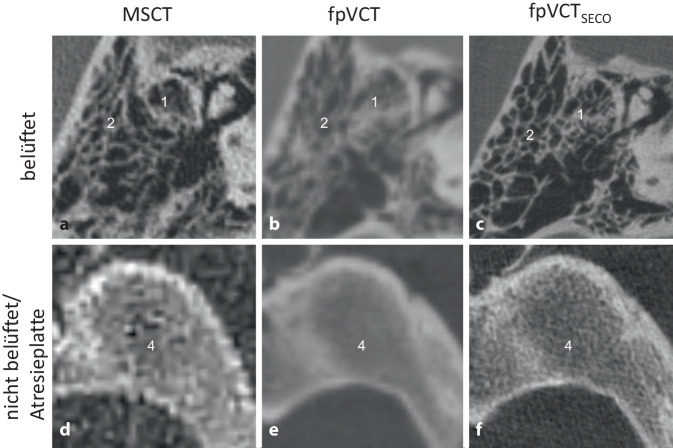
Visualisierung der Belüftung der Mastoidzellen. Ein repräsentatives Bild mit MSCT (600 µm Schichtdicke) wird in **a** gezeigt, eine Darstellung von fpVCT (466 µm Schichtdicke) in **b** und fpVCT_SECO_ (99 µm Schichtdicke) in **c**. **d**–**f** zeigen eine nicht belüftete/atresierte Platte. *1* belüftete Mastoidzellen, *2* knöcherne Atresie des äußeren Gehörgangs, *4* Mastoidatresieplatte, *MSCT* Multislice-Computertomographie, *fpVCT* „flat-panel volume computed tomography“, *fpVCT*_*SECO*_ sekundäre Rekonstruktionen der fpVCT

Ein fehlender äußerer Gehörgang wurde in allen Bildgebungsmodalitäten festgestellt. Einzelne Mastoidzellen waren jedoch in der niedrig auflösenden Bildgebung wesentlich schwieriger abzugrenzen. Eine genaue Visualisierung, insbesondere bei der Unterscheidung zwischen einem vollständig atretischem Knochen und verbleibenden belüfteten Mastoidzellen, war nur mit der fpVCT_SECO_ möglich (Abb. 4).

## 3-D-Visualisierung der Gehörknöchelchen

**Abb. 5:**
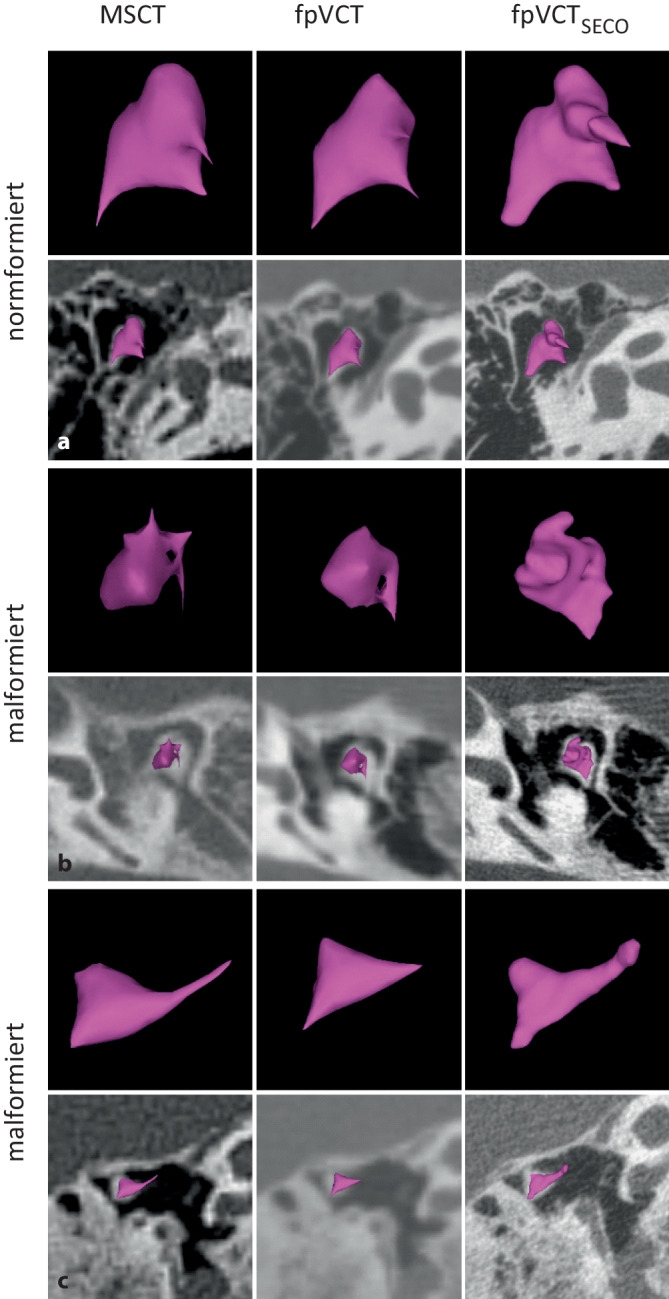
3‑D-Darstellung des Malleus-Incus-Komplexes in der Planungssoftware OTOPLAN® mittels MSCT (600 µm Schichtdicke), fpVCT (466 µm Schichtdicke) und fpVCT_SECO_ (99 µm Schichtdicke). Die Komplexe sind jeweils isoliert und mit einer überlagerten axialen Ebene dargestellt. **a** zeigt einen normal konfigurierten Komplex. Ein mäßig fehlgebildeter Komplex ist in **b** und ein stark fehlgebildeter Komplex in **c** dargestellt. *MSCT* Multislice-Computertomographie, *fpVCT* „flat-panel volume computed tomography“, *fpVCT*_*SECO*_ sekundäre Rekonstruktionen der fpVCT

Um die 3‑D-Visualisierung der verschiedenen Bildgebungsmodalitäten zu vergleichen, wurde der Malleus-incus-Komplex mit dem automatischen Werkzeug der chirurgischen Planungssoftware segmentiert. Wie Abb. 5 zeigt, war die Visualisierung des Komplexes mit fpVCT_SECO_ im Vergleich zu MSCT wesentlich detaillierter und anatomisch korrekt segmentiert. Die MSCT stellte die missgebildeten Komplexe nicht zufriedenstellend dar.

### Vergleich der Grading-Scores

**Abb. 6:**
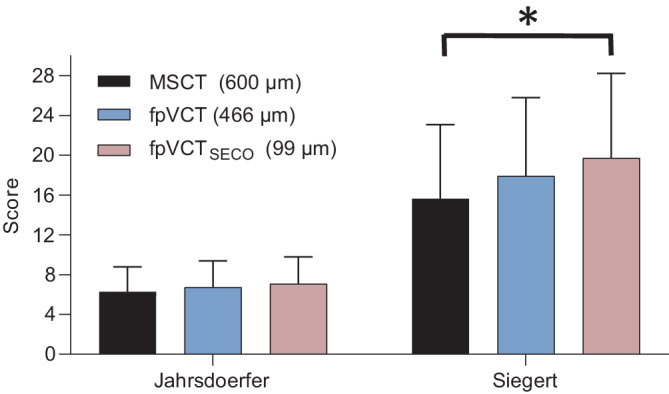
Analyse der CT(Computertomographie)-Grading-Scores bei Auralatresie in verschiedenen radiologischen Einstellungen und Modalitäten. *Links* ist der Jahrsdoerfer-Score, *rechts* der Siegert-Score dargestellt. Die Anzahl der untersuchten Felsenbeine betrug *n* = 7. Die Bewertung der Grading-Scores erfolgte durch 2 Gutachter. Unterschiede zwischen den angegebenen Kohorten sind als Signifikanz angegeben, *p = 0,05. *MSCT* Multislice-Computertomographie, *fpVCT* „flat-panel volume computed tomography“, *fpVCT*_*SECO*_ sekundäre Rekonstruktionen der fpVCT

Die Werte für die 3 Bildgebungsmodalitäten sind in Abb. 6 dargestellt. Der Mittelwert des Jahrsdoerfer-Scores (Referenzbereich: 0–10 Punkte) betrug für MSCT 6,3 (SD [„standard deviation“]: 3,4), für fpVCT 6,7 (SD: 3,2) und für fpVCT_SECO_ 7,1 (SD: 2,9). Der Vergleich der Mittelwerte ergab keine signifikanten Unterschiede. Auch zwischen den Gruppen gab es keine signifikanten Unterschiede. Bei der Auswertung des Siegert-Scores betrug der Mittelwert (Referenzbereich: 0–28 Punkte) für MSCT 16,2 (SD: 11,9), für fpVCT 18,6 (SD: 10,7) und für fpVCT_SECO_ 20,4 (SD: 9,4). Der Vergleich der Mittelwerte ergab einen signifikanten Unterschied (*p* = 0,0125). Auch der Unterschied zwischen der Auswertung mittels fpVCT_SECO_ und MSCT war signifikant (Mittelwert 4,1; *p* = 0,0125).

## Diskussion

Bei kongenitalen Auralatresien besteht eine große Herausforderung in der adäquaten präoperativen Beurteilung der Pathologie, die für die richtige Entscheidung über die effektivste Operationsart und die beste Hörverbesserung entscheidend ist. Dies erfordert generell und insbesondere bei jungen Patienten eine solide radiologische Diagnostik. Die vorgestellten Daten zeigen, dass die präoperative Bildgebung mit sekundären Rekonstruktionen der „flat-panel volume computed tomography“ (fpVCT_SECO_) die systematische Analyse der individuellen Anatomie, einschließlich Anomalien des Mittelohrs erleichtert. Darüber hinaus kann die 3‑D-Visualisierung mit otologischen Planungssoftware die anatomische Genauigkeit verbessern.

Die konventionelle MSCT-Bildgebung (600 µm Schichtdicke) wurde mit fpVCT-Bildgebung (466 µm Schichtdicke) und deren sekundärer Rekonstruktionen (fpVCT_SECO_; 99 µm Schichtdicke) verglichen. Dazu wurden die jeweiligen Strukturen zur Bestimmung Jahrsdoerfer- und Siegert-Grading-Scores untersucht und ein neuer, selbst entwickelter Score („ossicle score“) berechnet. Die Identifizierung der Gehörknöchelchen ist bei der radiologischen Beurteilung von Auralatresien von entscheidender Bedeutung. Die Substrukturen des Stapes, des Incudostapedialgelenks und des Malleus-Incus-Komplexes konnten mit dem fpVCT_SECO_ am genauesten identifiziert werden, wie in Tab. 2 dargestellt. Insbesondere zeigte sich, dass der Stapes nur mit dem fpVCT_SECO_ (Abb. 1) in seiner Gesamtheit erkennbar und vom umgebenden Gewebe abgegrenzt war, nicht aber mit dem fpVCT und MSCT, wo nur rudimentäre Fragmente des Stapes teilweise sichtbar waren. Eine Unterscheidung zwischen normalem und fehlgebildetem Stapes war bei diesen beiden Modalitäten oft nicht möglich. Dies ist wichtig, da der Stapes mit 2 Punkten im Jahrsdoerfer- und 4 Punkten im Siegert-Score die wichtigste Struktur des Jahrsdoerfer- und eine der wichtigsten im Siegert-Score darstellt. Die verbesserte Bildgebung gewinnt auch an klinischer Relevanz, da der Stapes eine bevorzugte Lokalisation für die Platzierung des sog. „floating mass transducer“ (FMT) der „vibrant soundbridge“ (VSB) bei Auralatresie ist und das Vorhandensein eines Stapes mit der initialen postoperativen Schwelle für die versorgte Sprachempfindung („aided speech reception“) bei Patienten mit einer Auralatresie korreliert [11]. Daher kann ein vorhandener, missgebildeter oder fehlender Stapes eine Orientierungshilfe für die Planung der Position des FMTs sein.

Darüber hinaus erfordert die Planung eines otochirurgischen Eingriffs eine präzise und sorgfältige präoperative Beurteilung weiterer anatomischen Strukturen [1], die durch die fpVCT_SECO_ verbessert wird (Abb. 2, 3 und 4). Ein abnormaler Verlauf des N. facialis schließt einen Eingriff nicht zwangsläufig aus, und es können trotzdem gute audiologische Ergebnisse erzielt werden. Das Risiko von Komplikationen ist in solchen Situationen jedoch erhöht [10, 23]. Die Identifizierung und Abgrenzung des N. facialis wurde mit der fpVCT_SECO_ verbessert, was die Sicherheit des geplanten chirurgischen Eingriffs erhöhen könnte. Der Einsatz der fpVCT_SECO_ kann auch die korrekte Indikationsstellung und die Planung des Eingriffs verbessern und dazu beitragen, Kontraindikationen wie die Obstruktion des ovalen Fensters durch einen abweichenden N. facialis vorherzusagen [23].

Die Daten der verschiedenen Bildgebungsmodalitäten wurden zusätzlich mit dem automatischen Segmentierungswerkzeug der otologischen Planungssoftware OTOPLAN® visualisiert, um den Malleus-Incus-Komplex in 3D darzustellen (Abb. 5). Die bildgebenden Eigenschaften dieser Struktur, insbesondere in missgebildeter Konfiguration, wurden mit fpVCT_SECO_ zuverlässig dargestellt. Im Gegensatz dazu führte die Verwendung von MSCT und fpVCT eher zu zusammengeklebt wirkenden quaderformartigen Objekten (Abb. 5), die nicht der Realität entsprachen. Dies ist auf das schwellenwertbasierte Softwareverfahren zurückzuführen. Bildgebende Verfahren mit geringerer Auflösung haben größere Schwellenbreiten und, insbesondere an den Rändern, einen geringeren Kontrast zum umgebenden Gewebe. Infolgedessen kann die Segmentierung in niedrig aufgelösten Bildern Strukturen in 3D nur unzureichend darstellen. Dennoch ist eine genaue 3D-Darstellung noch nicht möglich. Insbesondere an den äußeren Grenzen des Komplexes werden trotz der verbesserten Bildgebung mit fpVCT_SECO_ bestimmte Bereiche noch nicht erfasst. Allerdings könnte die fpVCT_SECO_ in Zukunft die Möglichkeit bieten, den Stapes in otologischen Softwarelösungen automatisch zu segmentieren, was derzeit aufgrund der relativ geringen Auflösung in der klinischen Bildgebung nicht möglich ist. Die Kombination von fpVCT_SECO_ und der fortschrittlichen Software könnte helfen, die otologischen Pathologien genauer zu klassifizieren und damit auch die Sicherheit sowie den Erfolg der Therapie zu erhöhen.

Die in dieser Studie eingeschlossenen Patienten mit einer Auralatresie wiesen ein breites Spektrum an Schweregraden auf, mit Jahrsdoerfer-Scores von 3–9 (Referenzwert: 0–10) und Siegert-Scores von 5–26 (Referenzwert: 0–28). Die Berechnung der Grading-Scores ergab einen signifikanten Unterschied im Siegert-Score zwischen den höheren Scores, die mit fpVCT_SECO_ ermittelt wurden, und den Scores, die für dieselben Felsenbeine mit MSCT und fpVCT ermittelt wurden (Abb. 6). Unter der Annahme, dass die fpVCT_SECO_ die tatsächliche Anatomie der untersuchten bildgebenden Verfahren am genauesten wiedergibt, könnte spekuliert werden, dass die Indikation für die Art der vorgeschlagenen Operation überdacht werden könnte. Dies gilt insbesondere für schwere Fälle einer Auralatresie. In einigen dieser Fälle würde möglicherweise ein höherer Score eine chirurgische Intervention rechtfertigen, da neuere Veröffentlichungen darauf hindeuten, dass ein Jahrsdoerfer-Score über 3 bereits zu einem hervorragenden audiologischen Ergebnis führen kann [5]. Mit anderen Worten: In Grenzfällen wäre es möglich, dass ein Patient auf der Grundlage der MSCT nicht als guter Kandidat für eine Atresie-Operation oder ein Mittelohrimplantat angesehen wird, wohl aber auf der Basis der Bildgebung mit fpVCT_SECO_.

Insgesamt ist die CT-Bildgebung auch bei pädiatrischen Patienten gerechtfertigt, um folgende Aspekte abzuklären und zu berücksichtigen:

(i) Exakte Diagnosestellung mit Bestimmung von Art und Ausmaß der Auralatresie.
(ii) Beurteilung möglicher begleitender kraniofazialer Anomalien.
(iii) Erstellung eines fundierten Behandlungsplans (der einen chirurgischen Eingriff einschließen kann, wobei die beste Vorgehensweise zur Minimierung potenzieller Risiken und Herausforderungen im Voraus festgelegt wird) und
(iv) Durchführung von Verlaufskontrollen (um Veränderungen im Laufe der Zeit zu überwachen und die Behandlung ggf. anzupassen).

Es ist jedoch zu beachten, dass es sich bei der hier untersuchten Population hauptsächlich um Jugendliche und junge Erwachsene handelt, bei denen eine CT-Untersuchung in der Regel problemlos im Wachzustand durchgeführt werden kann. Dagegen erscheint es bei Kleinkindern sinnvoll, die CT-Untersuchung in Kurznarkose oder Sedierung durchzuführen, um das Risiko für Bewegungsartefakte zu minimieren. Da bei Patienten in diesem jungen Alter aber ohnehin immer auch eine präoperative Hördiagnostik mit beispielsweise einer Hirnstammaudiometrie („brainstem evoked response audiometry“, BERA) zwingend erforderlich ist, könnten in solchen Fällen sowohl die Hördiagnostik als auch die Bildgebung in einer gemeinsamen Kurznarkose/Sedierung erfolgen und diese somit vertretbar machen. Es sollte jedoch weiterhin sorgfältig und individuell abgewogen werden, ob diagnostische Untersuchungen mit ionisierender Strahlung im Kindesalter wirklich notwendig sind oder auch zu einem späteren Zeitpunkt durchgeführt werden können. Dies gilt insbesondere dann, wenn ein operativer Eingriff erst zu einem späteren Zeitpunkt, etwa in der Adoleszenz oder im frühen Erwachsenenalter, geplant ist. Idealerweise sollten daher junge Patienten mit Auralatresie nur dann einer CT-Bildgebung unterzogen werden, wenn die Eltern ein klares Verständnis der Pathologie und ein daraus resultierendes Interesse und den Wunsch nach einer möglichen Operation haben.

### Limitationen der Studie

Da Patienten mit Auralatresie häufig mit externer Bildgebung überwiesen werden, ist darauf hinzuweisen, dass die MSCT-Bildgebungsdaten mit unterschiedlichen Geräten, jedoch mit ähnlicher Schichtdicke aufgenommen wurden. Dies könnte die Auswertung beeinflusst haben. Darüber hinaus gab es teilweise eine zeitliche Differenz zwischen den MSCT- und fpVCT-Scans, was möglicherweise zu einer Verzerrung, insbesondere in Bezug auf die Ventilation der Mastoidzellen, geführt haben könnte.

Aus strahlenhygienischen Gründen wird insbesondere bei jungen Patienten darauf geachtet, möglichst nur eine CT-Aufnahme durchzuführen, was die Stichprobengröße einschränkt (7 Felsenbeine von 6 Patienten), da häufig nicht alle 3 untersuchten Bildgebungsmodalitäten desselben Felsenbeins für einen Vergleich zur Verfügung stehen. Frühere radiologische Studien, die beispielsweise die cochleäre Länge („cochlear duct length“; CDL) in Cochlea untersucht haben, konnten jedoch zeigen, dass eine solche Anzahl an Patienten adäquat ist, um eine suffiziente statistische Aussagekraft zu erreichen [14, 18, 20, 21]. Überdies zeigen die Ergebnisse nichtsdestotrotz, dass eine höher aufgelöste Bildgebung die Anatomie genauer darstellt und zuverlässigere Indikationen liefert, insbesondere bei ausgeprägter Malformation.

## Fazit für die Praxis

- Die Studie zeigt, dass Bildgebungen mit höherer Auflösung, wie die sekundären Rekonstruktionen der „flat-panel volume computed tomography“ (fpVCT_SECO_) mit 99 µm Schichtdicke, kongenitale Auralatresien genauer darstellen können.
- Dies ist klinisch besonders wichtig für die Planung hörverbessernder Operationen.
- Besonders bei pädiatrischen Patienten, die eine optimale Versorgung benötigen, bietet dies die beste Beurteilung der Pathologie. Daher wird empfohlen, auch bei Auralatresien fpVCT-Bildgebungen mit sekundären Rekonstruktionen durchzuführen.
